# Seeding Activity of Skin Misfolded Tau as a Biomarker for Tauopathies

**DOI:** 10.21203/rs.3.rs-3968879/v1

**Published:** 2024-03-04

**Authors:** Zerui Wang, Ling Wu, Maria Gerasimenko, Tricia Gilliland, Steven A. Gunzler, Vincenzo Donadio, Rocco Liguori, Bin Xu, Wen-Quan Zou

**Affiliations:** Case Western Reserve University School of Medicine; North Carolina Central University; Case Western Reserve University; Case Western Reserve University; University Hospitals Cleveland Medical Center: UH Cleveland Medical Center; IRCCS Institute of Neurological Sciences of Bolgna: IRCCS Istituto Delle Scienze Neurologiche di Bologna; IRCCS Institute of Neurological Sciences of Bologna: IRCCS Istituto Delle Scienze Neurologiche di Bologna; North Carolina Central University; First Affiliated Hospital of Nanchang University

**Keywords:** Tauopathies, Alzheimer’s disease, seeding activity, tau, real-time quaking-induced conversion (RT-QuIC), skin

## Abstract

**Background:**

Tauopathies are a group of age-related neurodegenerative diseases characterized by the accumulation of pathologically phosphorylated tau protein in the brain, leading to prion-like propagation and aggregation. They include Alzheimer’s disease (AD), progressive supranuclear palsy (PSP), corticobasal degeneration (CBD), and Pick’s disease (PiD). Currently, reliable diagnostic biomarkers that directly reflect the capability of propagation and spreading of misfolded tau aggregates in peripheral tissues and body fluids are lacking.

**Methods:**

We utilized the seed-amplification assay (SAA) employing ultrasensitive real-time quaking-induced conversion (RT-QuIC) to assess the prion-like seeding activity of pathological tau in the skin of cadavers with neuropathologically confirmed tauopathies, including AD, PSP, CBD, and PiD, compared to normal controls.

**Results:**

We found that the skin prion-SAA demonstrated a significantly higher sensitivity (75–80%) and specificity (95–100%) for detecting tauopathy, depending on the tau substrates used. Moreover, increased tau-seeding activity was also observed in biopsy skin samples from living AD and PSP patients examined. Analysis of the end products of skin-tau SAA confirmed that the increased seeding activity was accompanied by the formation of tau aggregates with different physicochemical properties related to two different tau substrates used.

**Conclusions:**

Overall, our study provides proof-of-concept that the skin tau-SAA can differentiate tauopathies from normal controls, suggesting that the seeding activity of misfolded tau in the skin could serve as a diagnostic biomarker for tauopathies.

## INTRODUCTION

The deposition of disease-associated tau aggregates in the brain is a characteristic feature of tauopathies, including Alzheimer’s disease (AD), progressive supranuclear palsy (PSP), corticobasal degeneration (CBD), and Pick’s disease (PiD) [1]. These conditions share a common pathogenesis involving the seeding and propagation of misfolded and phosphorylated isoforms of the tau protein in the brain, reminiscent of the infectious prion protein (PrP^Sc^ or prion) in prion diseases (PrD) [2]. The human brain expresses six tau isoforms, resulting from the combination of three or four microtubule-binding repeats (3R or 4R tau) and 0–2 N-terminal inserts (0N, 1N, or 2N tau) [3]. Different tauopathies exhibit variations in the composition of these tau isoforms, with AD displaying both 3R and 4R isoforms, PiD primarily containing 3R isoforms, and PSP and CBD characterized by the accumulation of 4R tau assemblies. These differences in tau isoform composition are proposed to be associated with the presence of distinct strains of neurotoxic tau aggregate conformers [4]. Currently, the definitive diagnosis of tauopathies relies on the availability of brain tissue obtained via biopsy or autopsy for the detection of tau pathology and phosphorylated tau. However, these methods are highly invasive or often too late in the disease process. Recent advancements in brain molecular imaging and highly sensitive immunoassays of phosphorylated and total tau (p-tau and t-tau) in plasma and cerebrospinal fluid (CSF) have enabled early and reliable diagnosis of AD in living patients [5], but they have limitations such as invasiveness or high cost. Newly developed single molecular immunoassay (Simoa) is highly sensitive and specific in detection of the blood biomarkers, but Simoa is unaffordable to the majority of patients, especially those in the developing countries.

The seed-amplification assay (SAA) technology, utilizing real-time quaking-induced conversion (RT-QuIC) or protein misfolding cyclic amplification (PMCA), offers an ultrasensitive approach to identify disease-specific biomarkers in easily accessible specimens for early diagnosis and disease progression assessment [6–8]. This technology has been used to detect prions and other prion-like misfolded proteins such as α-synuclein in body fluids and peripheral tissues of PrD and PD by assessing the seeding activity (SA) of misfolded proteins [9–15]. RT-QuIC has also been reported to detect tau-SA in the brain tissues of deceased individuals with tauopathies [16–20]. However, there have not been any reports on the application of tau-SAA in the body fluids and peripheral tissues of patients with AD to date although there was a report about tau RT-QuIC detection of CSF from PSP and CBD [19].

In the current study, we investigated tau seeding activity in autopsy scalp skin samples from individuals with neuropathologically confirmed AD, PSP, CBD, PiD, and normal controls using RT-QuIC with truncated human tau fragments as substrates. We found that misfolded tau from AD and other tauopathies like PSP and CBD, but not from normal controls and PiD, selectively seeded the 4RCF substrate (equivalent to the human 4R tau fragment, K18CFh) [16, 20] with a sensitivity of 80.5% and a specificity of 95.4%. Similarly, the 3RCF substrate (equivalent to the human 3R tau fragment, K19CFh) [18, 20]showed a sensitivity of 77% and a specificity of 92%. Biopsied skin samples from individuals with AD, PSP, and controls also exhibited high diagnostic efficacy. Furthermore, the study demonstrated that skin-derived misfolded tau aggregates from AD, containing a mix of 4R and 3R tau, and from PSP and CBD, with solely 4R tau, could be successfully amplified using either 4RCF or 3RCF substrates. In contrast, skin misfolded tau aggregates from PiD, exclusively composed of 3R tau, showed amplification only with the 3RCF substrate, not with 4RCF. This study represents the first application of SAA methodology to both post-mortem and biopsied cutaneous specimens and highlights the potential of skin tau-SAA as a novel biomarker for the diagnosis of tauopathies.

## RESULTS

### Phosphorylated-tau is detectable in skin samples of participants with tauopathies

We first determined whether the pathologically phosphorylated tau is detectable in the skin samples of participants with tauopathies by western blotting. Skin homogenates from deceased individuals with AD (n = 5), PSP (n = 4), CBD (n = 4), PiD (n = 4) and NC (n = 4) were examined by western blotting with antibodies directed against phosphorylated tau (Anti-pT231 and Anti-pS396). P-tau231 and p-tau396 epitopes were previously identified from a systematic p-tau antibody screening in differential detection with AD and control brains [21]. Both Anti-pT231 (Fig. S1A) and Anti-pS396 (Fig. S1b) antibodies revealed protein bands migrating at between approximately 25 and 100 kDa in all AD, 1 of 4 PSP, 2 of 4 CBD, and 1 of 4 PiD skin samples (Fig. S1). These bands are expected to represent truncated and full-length monomers as well as oligomers of phosphorylated tau.

In contrast, normal controls mainly displayed a band migrating at about 100 kDa without low molecular weight bands. The levels of phosphorylated tau in the skin of AD participants were significantly elevated compared to those of other tauopathies and normal controls (Fig. S1D, 1E), as evidenced by the quantitative analysis after normalized each sample by corresponding ß-actin migrating at around 50 kDa as a loading control (Fig. S1C). To determine the specificity of the protein bands and exclude the possibility that the bands might be from the non-specific reaction of the secondary antibody, we probed the blots with the secondary antibody only in the absence of the primary antibody (Fig. S2). The absence of detectable bands following a long ECL exposure (90 minutes) suggested that the bands identified by phospho-tau antibodies were indeed specific. The results revealed that the levels of the skin phosphorylated tau were significantly greater in AD than in other tauopathies and controls. Moreover, to verify that the bands identified are indeed tau proteins, we employed two distinct anti-tau antibodies: tau 46 (Invitrogen, MA), which binds to tau residues spanning 404 to 411 (Fig. S3A), and 43D (Biolegend, CA), targeting tau residues 1 to 100 (Fig. S3B). These antibodies, designed to detect total tau in samples, specifically recognized the protein bands in the same collection of skin samples. Notably, the pattern of these bands differed from those identified by phosphorylated tau antibodies, further substantiating that the tau proteins we detected are indeed specific.

### Skin tau-seeding activity can specifically differentiate AD and non-AD tauopathies from normal controls and PiD cases using 4RCF as the substrate

In contrast to PrP in PrD and α-synuclein in PD, the tau molecule in the human brain exhibits 6 isoforms, of which there are 3 isoforms with 4 microtubule-binding repeats (4R tau) and 3 with three repeats (3R tau), resulting from alternative mRNA splicing. In order to find an appropriate tau substrate for the seed-amplification assay (SAA) of skin tau (sTau-SAA) with RT-QuIC, based on our recent finding with autopsy brain tissues from cadavers with AD and other non-AD tauopathies [20], we first examined 4 types of tau substrates including 2 full-length tau isoforms (2N3R/2N4R) and 2 truncated tau fragments consisting of 4RCF (cysteine-free, equivalent to K18CFh)/3RCF (equivalent to K19CFh) using autopsy skin samples from AD cadavers. 4RCF represents the aggregation-prone core sequences of the 4R tau isoforms [20, 21]. Correspondingly, 3RCF fragment represents the aggregation-prone core sequences of the 3R tau isoforms. The only difference between 4RCF and 3RCF is that 3RCF lacks R2 region. Compared to the full-length tau, the truncated tau substrates-based RT-QuIC revealed higher endpoint fluorescence readings (exceeding 100,000 RFU) and shorter lag phases (less than 20 hours) (Fig. S4). Then we compared tau-SA of brain and skin samples from AD and non-AD controls using two truncated tau fragments as substrates. AD skin samples exhibited a slightly extended lag phase compared to AD brain samples when assessed with both 4RCF (Fig. S6A) and 3RCF (Fig. S6B) tau substrates. Furthermore, both brain and skin samples from non-AD controls demonstrated significantly weaker ThT fluorescence intensity compared to the AD samples when analyzed with the two types of tau substrates. As a result, we used the two truncated tau fragments as the substrates for the following examinations in our study.

**Figure 1:**
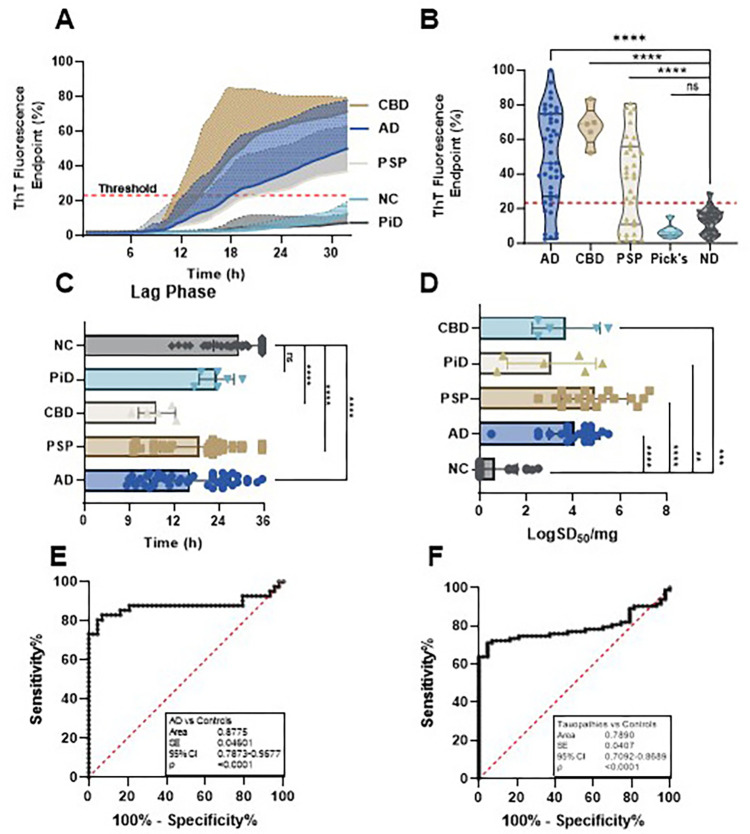
Tau-seeding activity of skin samples from patients with tauopathies using 4RCF-based RT-QuIC. (**A**) Kinetic curves displaying the mean and standard deviation (SD) of tau-SA over time of skin samples from CBD (n = 5), AD (n = 46), PSP (n = 33), PiD (n = 6) and NC (n = 43). (**B**) Scatter plot illustrating the distribution of tau-SA across different tauopathies detected in panel (**A**). (**C**) Lag phase, defined as the initial period before a significant increase in the ThT fluorescence curves. (**D**) The end-point dilution analysis of quantitative tau-SA of skin samples from tauopathies. The half of maximal SA (SD50) was determined by Spearman-Kärber analyses and is shown as log SD50/mg skin tissue. (**E**) Receiver operating characteristic (ROC) curve analysis comparing AD patients and control subjects, with an area under the curve (AUC) of 0.82. (**F**) ROC curve analysis comparing total tauopathies and control subjects, with an AUC of 0.79. ns: *p* > 0.05; **: *p* < 0.01; ***: *p* < 0.001; ****: *p* < 0.0001.

Using RT-QuIC with 4RCF truncated tau as the substrate, we analyzed autopsy skin samples from AD (n = 46), CBD (n = 5), PSP (n = 33), PiD (n = 6), and NC (n = 43). 4RCF-based RT-QuIC of skin tau-seeding activity (sTau-SA) exhibited that CBD cases had the highest ThT fluorescence intensity, followed by AD, PSP, and PiD (Fig. 1A, B). We also compared lag phases of sTau-SA in each type of tauopathy, the time period between the beginning of the RT-QuIC measurement and the time point at which the curves reflecting the ThT fluorescence started to increase in their ThT fluorescence. Consistent with the endpoint ThT values, CBD displayed the shortest lag phase, while PiD was the longest (Fig. 1C). Our 4RCF-based RT-QuIC assay of the skin samples from AD and non-AD tauopathies yielded a sensitivity of 80.49% and a specificity of 95.35%. To quantitate the sTau-SA of each type of tauopathy, we conducted endpoint titration of RT-QuIC assay of each skin sample from different tauopathies. The x-axis represents SD50 that referred to the 50% seeding activity. PSP samples exhibited the highest sTau-SA, followed by AD samples. In contrast, PiD skin samples displayed markedly lower tau-SA, as shown in Fig. 1D. This observed variation is likely attributable to the incompatibility between seed and substrate, particularly noting that PiD-derived misfolded tau is uniquely comprised of 3R tau, absent of 4R tau components. AUC analysis revealed an area value of 0.88 based on the comparison between AD and normal controls and 0.79 based on the comparison between tauopathies and normal controls (Fig. 1E, F).

### The sTau-SA of AD and all tauopathies can be specifically differentiated from that of normal controls using 3RCF as the substrate

**Figure 2:**
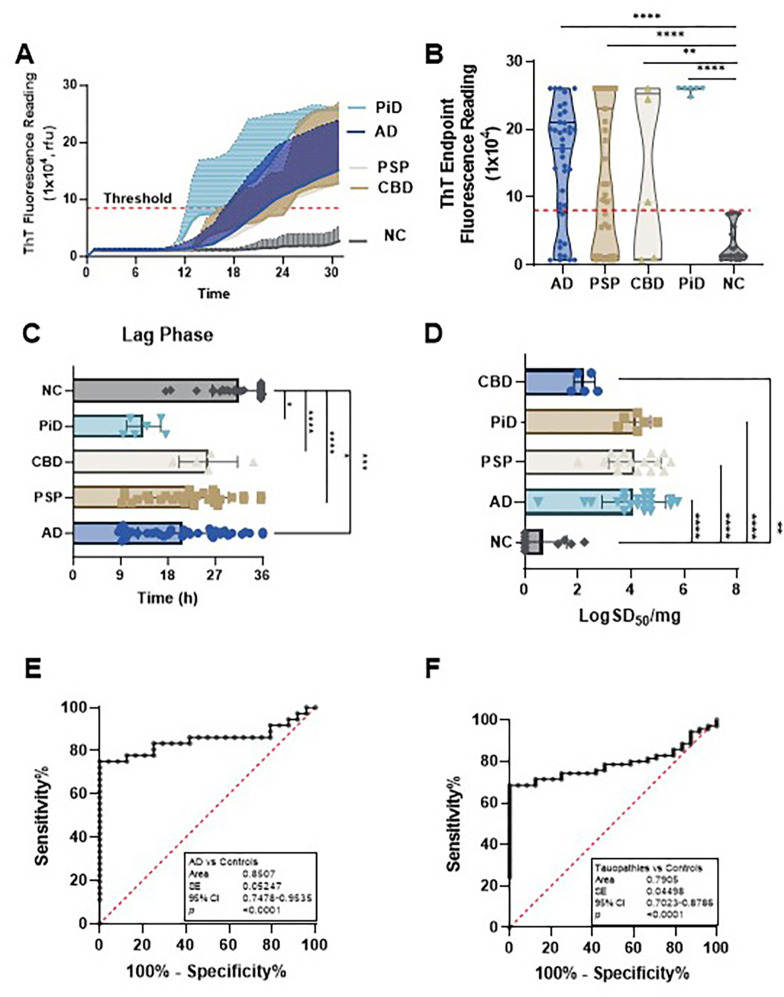
Tau-seeding activity of skin samples from patients with tauopathies using 3RCF-based RT-QuIC assay. (**A**) Kinetic curves displaying the mean and SD of tau-SA over time of skin samples from CBD (n = 5), AD (n = 46), PSP (n = 33), PiD (n = 6) and NC (n = 43). (**B**) Scatter plot illustrating the distribution of tau-SA across different tauopathies. (**C**) Lag phase, same as above, as the initial delay before the ThT fluorescence curves begin to rise. (**D**) The end-point dilution analysis of quantitative tau-SA of skin samples from tauopathies. The half of maximal SA (SD50) determined by Spearman-Kärber analyses is shown as log SD50/mg skin tissue. (**E**) ROC curve analysis comparing AD patients and control subjects, with an AUC of 0.77. (**F**) ROC curve analysis comparing total tauopathies and control subjects, with an AUC of 0.72. *: *p* < 0.05; **: *p* < 0.01; ***: *p* < 0.001; ****: *p* < 0.0001.

We next used 3RCF (K19)-based RT-QuIC assays to examine the same samples detected in 4RCF studies. The sTau-SA was significantly higher in tauopathies than in non-tauopathies, of which PiD was the highest, followed by AD, PSP, and CBD (Fig. 2A, B). We also compared their lag phases, which were inversely proportional to those of 4R tau: PiD exhibited the shortest lag phase, while CBD showed the longest lag phase (Fig. 2C). PiD and PSP demonstrated the highest seeding dose while CBD had the lowest one (Fig. 2D). In addition, the 3RCF-based skin tau RT-QuIC assay generated a sensitivity of 75% and a specificity of 100%, which was similar to that of 4RCF-based RT-QuIC in general. The AUC values (0.85 vs 0.79) of 3RCF-based RT-QuIC assay of skin tau were similar to those detected with 4RCF-based RT-QuIC shown above (Fig. 2E, F). Notably, in contrast to the 4RCF-based sTau-SA, 3RCF-based sTau-SA from PiD was the highest, in addition to differentiating other tauopathies from the normal controls.

### The sTau-SA is significantly elevated over an increase in the Braak staging

**Figure 3:**
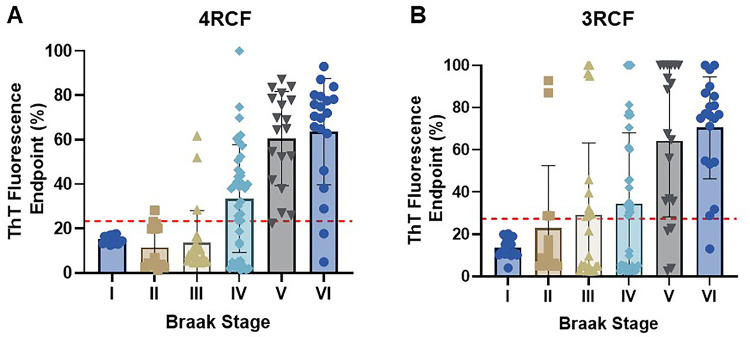
Tau-seeding activity of skin samples from AD and other tauopathies with different Braak stages. Scatter plot of tau-SA as a function of Braak stages through RT-QuIC assays with the substrate 4RCF (**A**) or 3RCF (**B**). ns: *p* > 0.05; ** *p* < 0.01; *** *p*< 0.001; ****p< 0.0001.

Based on Aβ/tau-pathology and Aβ/tau-positron emission tomography (PET) scan, the accumulation of Aβ/tau leading to clinical AD is a continuum process. To determine whether the skin tau-SA can reflect the severity or Braak staging in the AD brain, next we associated skin tau-SA with the Braak staging in autopsy brain tissues examined. Notably, when sTau-SA with both 4RCF and 3RCF served as a function of Braak staging, there was a clear association observed: the sTau-SA was significantly elevated upon an increase in the Braak staging (Fig. 3). Specifically, with the exception of stages I and II, the overall ThT intensity for 4RCF-based skin tau-SA was increased following advancing of the Braak stages, although no significant difference was noted between stages V and VI (Fig. 3A). A similar trend was observed for 3RCF, where the overall ThT intensity increased with the progression of Braak stage (Fig. 3B), yet no significant difference was found between stages V and VI or between stages III and IV.

We also explored whether variables such as age, gender, and post-mortem interval (PMI) could influence Tau-SA. We observed significant differences between males and females in both 4RCF (Fig. S6A, 6B) and 3RCF (Fig. S6C, 6D) assays. In the correlation analysis between ThT fluorescence intensity and age/PMI of the autopsied skin tissues, no correlation was found for 4RCF with either age (Fig. S6E) (r = 0.07247, p = 0.5047) or PMI (Fig. S6F) (r = −0.02187, p = 0.8406). For 3RCF, a slight positive correlation was observed between age and Tau-SA end-point ThT fluorescence, though this was not statistically significant (Fig. S6G) (r = 0.1042, p = 0.3366); similarly, a slight negative correlation was noted between PMI and ThT fluorescence (r = −0.1225, p = 0.2583) (Fig. S6H).

### The sTau-SA is significantly higher in PD and dementia with Lewy bodies than in multiple system atrophy and normal controls but it is still lower than that in AD

**Figure 4:**
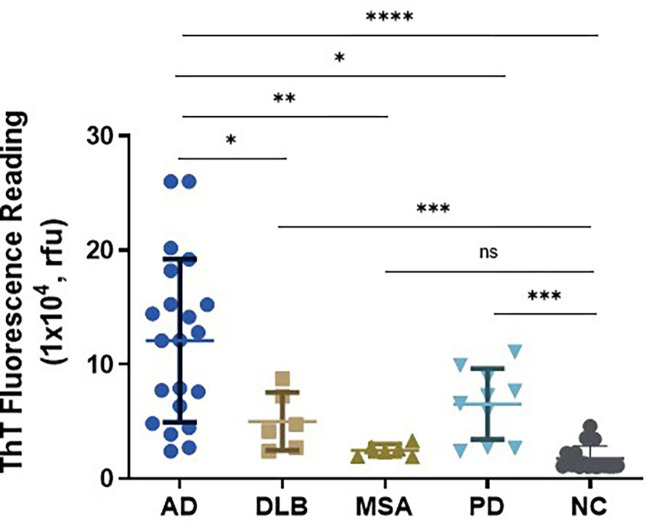
Tau-SAA of skin samples from participants with AD, synucleinopathies, and NC using 4RCF-based RT-QuIC assay. Scatter plot illustrating the distribution of tau-SA at the endpoint fluorescence readings across skin samples from 21 cases with AD, different synucleinopathies including 6 cases with DLB, 6 with MSA and 10 with PD as well as 17 NCs. *: *p* < 0.05, **: *p* < 0.001; ****: *p* < 0.0001.

Accumulation of tau aggregates in the brain has been observed in some of cases with synucleinopathies including PD, dementia with Lewy bodies (DLB), and multiple system atrophy (MSA) (22–26). Next, we further explored whether tau-SA can be detected in the skin of synucleinopathies by our sTau-SAA. Autopsy skin samples from AD (n = 21), PD (n = 10), MSA (n = 6), DLB (n = 6), and NC (n = 17) were examined by 4RCF-based tau-SAA. The ThT endpoint fluorescence intensity of sTau-SAA was dramatically higher in AD than in synucleinopathies, whereas the skin-tau fluorescence intensity was also significantly increased in synucleinopathies except for MSA than in the control group (Fig. 4), consistent with the previous observations that some of cases with synucleinopathies can have tau-pathology [10, 19, 37, 44, 48]. To determine the presence of tau pathology in skin samples from individuals with synucleinopathies, we performed western blotting on representative skin samples from PD (n = 7), DLB (n = 5), and MSA cases (n = 5). These samples were analyzed using anti-phospho-tau antibodies Anti-pT231 (Fig. S7A) and Anti-pS396 (Fig. S7B). We observed that 4 out of the 5 DLB samples exhibited positive bands in the range of 25–50 kDa. Notably, these 4 samples also had coexisting AD pathology as indicated in Table 1. In MSA samples, positive bands were also detected migrating at 25–50 kDa on the gels, although none of these cases had co-morbidities with confirmed tauopathies. However, tau tangles and plaques were detectable in their brain tissues (as shown in Table 1). Regarding the PD skin samples, 4 out of 7 cases displayed smeared bands that were indicative of positive phospho-tau detected by the two antibodies.

### Biopsy sTau-SA is significantly higher in tauopathies than in normal controls

**Figure 5:**
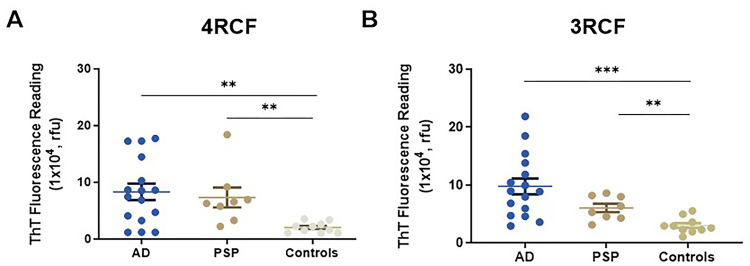
Examination of tau-SA in biopsied skin samples from patients with AD and PSP using 3RCF- or 4RCF-based RT-QuIC assay. The scatter plot displays the endpoint ThT fluorescence intensity for AD (n = 16), PSP (n = 8), and normal control samples (n = 10). **: *p* < 0.01; ***: *p* < 0.001.

We then used the above 4RCF- or 3RCF-based SAA (4RCF- or 3RCF-SAA) to examine biopsied skin samples from AD (n = 16), PSP (n = 8) and NCs (n = 10). Skin 4RCF-SA was significantly higher in AD than in normal controls [83091 ± 57912 (mean ± SD) vs 20490 ± 9307, p = 0.0026 < 0.005]; skin 4RCF-SA was also significantly greater in PSP than in normal controls (73360 ± 49625 vs 20490 ± 9307, p = 0.0043 < 0.005) (Fig. 5A). Similar to skin 4RCF-SA, 3RCF-SA was significantly higher in AD than in NCs [97511 ± 54115 vs 30090 ± 13657, p = 0.0008 < 0.001] and greater in PSP than in NCs (60449 ± 20492 vs 30090 ± 13657, p = 0.0017 < 0.005) (Fig. 5B). There were no significant differences in sTau-SA between AD and PSP cases with both substrates (Fig. 5). This observation implied the potential for sTau-SA to serve as an antemortem diagnostic biomarker to differentiate tauopathies from normal controls. The individual clinical data are listed in Tables 2 and 3.

### ThT fluorescence levels of sTau-SAA end-point correlate with dot-blot intensity of captured tau aggregate of the RT-QuIC end products

**Figure 6:**
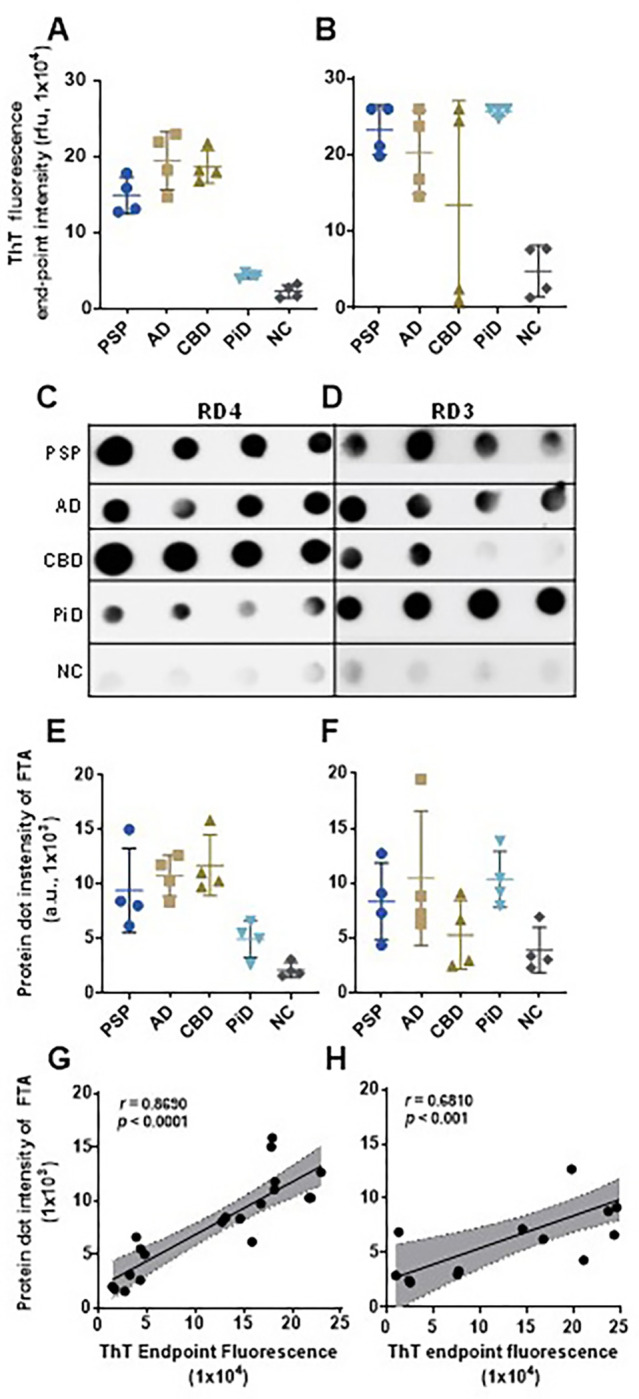
Characterization of RT-QuIC end products of skin tau from tauopathies using Filter-Trap Assay (FTA) probed with anti-3R (RD3) and anti-4R (RD4) tau antibodies. Scatter plots of ThT fluorescence values skin-tau RT-QuIC end products of selected samples from PSP (n = 4), AD (n = 4), CBD (n = 4), PiD (n = 4) and NC (n = 4), with (**A**) 4RCF and (**B**) 3RCF substrates. FTA assays of 3RCF-/4RCF-based RT-QuIC end products of skin samples from different tauopathies including PSP, AD, CBD, PiD and normal controls (4 cases for each group) probed with RD4 (**C**) or RD3 (**D**) antibodies. Densitometric quantification of density of FTA-dot blotting with 4RCF- (**E**) and 3RCF (**F**) -based RT-QuIC end products from panels (**C**) and (**D**). Correlation analysis between FTA-trapped protein dot intensity and skin tau-SA of 4RCF- (**G**)/3RCF(**H**) -based RT-QuIC end products.

To determine whether ThT fluorescence levels reflecting sTau-SA represent the formation of skin tau-seeded aggregates, we correlated the end-point ThT fluorescence levels with the dot-blot intensity of tau aggregates captured by a filter-trap assay (FTA) (Fig. 6). After obtaining the end point ThT fluorescence levels of sTau-SA of 4 cases each from AD, PSP, CBD, PiD and NC with 4RCF or 3RCF as the substrate (Fig. 6A, B), we then ran FTA with their corresponding end products, followed by probing the dot-blots with anti-4R tau antibody (RD4) (Fig. 6C) and anti-3R antibody (RD3) (Fig. 6D). The semiquantitative densitometric scanning of protein dot intensity on the dot-blots revealed that similar to ThT fluorescence levels, the intensity of tau aggregates captured by FTA on the blots was significantly higher in tauopathies than in normal controls by both RD4 (Fig. 6E) and RD3 antibodies (Fig. 6F). Correlation analyses demonstrated that the intensity of the trapped aggregates from the end products correlated positively with the ThT fluorescence levels (r = 0.86 for 4R, r = 0.68 for 3R) (Fig. 6G, H).

### Transmission electron microscopy of 4RCF- or 3RCF-based RT-QuIC end products displays the protofibril-like structures

**Figure 7:**
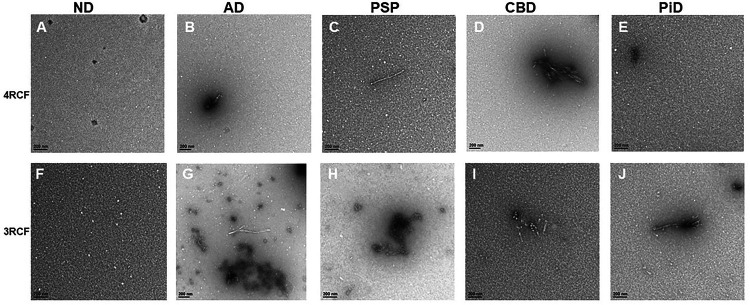
Transmission electron microscopy of SAA end products of skin misfolded tau from AD, other tauopathies and normal controls. Panels (**A**) through (**E**) show the representative images of tau 4RCF-based RT-QuIC with normal controls (NC, **A**), AD (**B**), PSP (**C**), CBD (**D**) and PiD (**E**). Panels (**F**) through (**J**) exhibit the representative images of tau 3RCF-based RT-QuIC with normal controls (NC, **F**), AD (**G**), PSP (**H**), CBD (**I**) and PiD (**J**). Scale bars: 200 nm.

While our FTA apparently was able to detect captured tau aggregates, to further determine the morphology of the amplified skin tau aggregates we performed transmission electron microscopy (TEM) of the RT-QuIC end-products of skin misfolded tau from 3 cases each of AD, PSP, CBD, PiD and normal controls (NC) with either 4RCF or 3RCF as the substrate (Fig. 7). For 4RCF, TEM revealed that except for PiD and NC (Fig. 7E), other end-products had a small number of protofibril-like structures. For 3RCF, while NC showed only oligomer-like structure (Fig. 7F), protofibrils were detectable in the end-product of tau-RT-QuIC of cases with all tauopathies by TEM (Fig. 7G through 7J).

### The end products of 4RCF- and 3RCF-SAA exhibit different patterns of resistance to proteinase K digestion

**Figure 8:**
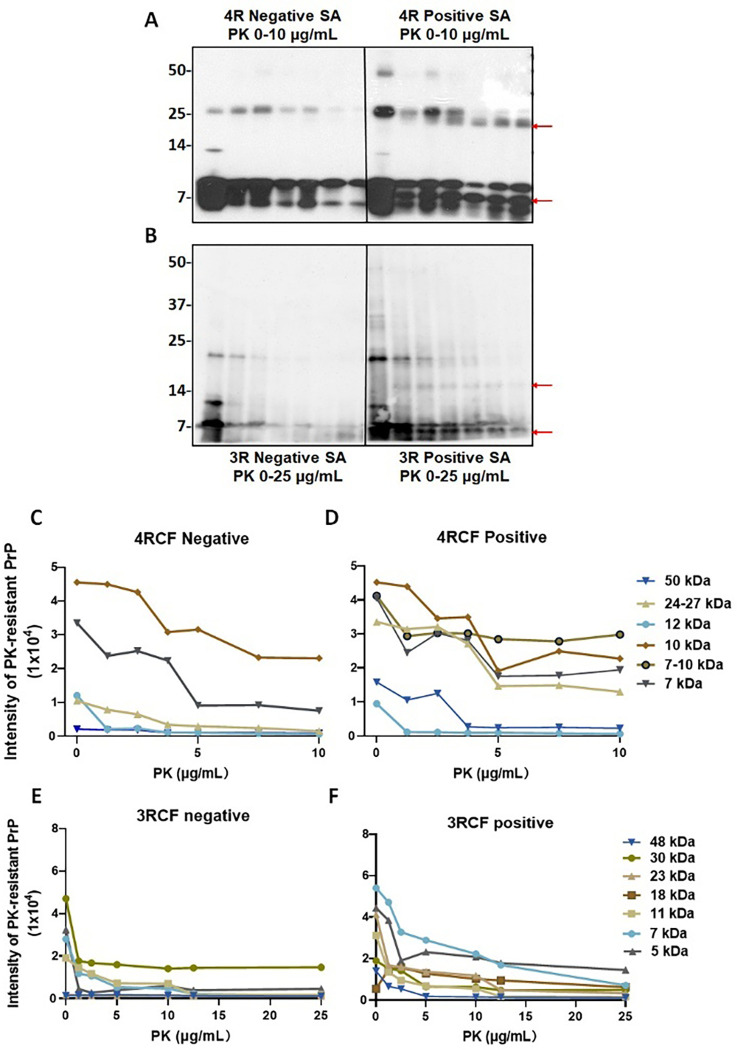
PK-resistance of the end products of 4RCF-/3RCF-based tau RT-QuIC assay of skin samples from AD and controls. Western blotting of PK titration of 4RCF- (**A**) and 3RCF (**B**) -based RT-QuIC end product of skin samples from AD and non-AD controls. Probed with RD3 and RD4 antibodies against 3R or 4R tau isoforms, respectively. Quantitative analysis of intensity of PK-resistant tau fragments by densitometry at different molecular weights for 4R (**C, D**) and 3R (**E, F**) substrates-based RT-QuIC end products.

The pattern of protein aggregates from the skin tau RT-QuIC end products to proteinase K (PK) digestion has been widely believed to reflect the conformational properties of misfolded proteins examined. Since the small fragments of 3RCF tau lower than 7 kDa were more difficult to digest by PK, we next performed the titration of varied PK concentrations ranging from 0, 1.25 μg/mL, 2.5 μg/mL, 3.75 μg/mL, 5 μg/mL, 7.5 μg/mL, to 10 μg/mL for the 4R (Fig. 8A) and 0, 1.25 μg/mL, 5 μg/mL, 10 μg/mL, 12.5 μg/mL and 25 μg/ml for the 3R tau (Fig. 8B) SAA end products of skin tau from AD and non-AD subjects. Without PK treatment, the end products of 4RCF-based RT-QuIC of skin tau from non-AD exhibited 3 protein bands by the RD4 tau antibody, migrating at approximately 25–26 kDa, 12–14 kDa, and 7–10 kDa (Fig. 8A). The short exposure of our blots showed that the 7–10 kDa bands actually consisted of 7 kDa and 10 kDa proteins. As a result, the above four bands could represent the trimer and dimer of a 7 kDa band as well as the monomers of a full-length 4RCF (~ 12 kDa) and a truncated 4RCF (~ 7–10 kDa), respectively. Of them, the two lower monomeric bands were predominant, whereas the top dimeric and trimeric tau bands were underrepresented, accounting for less than 1–2% of total tau (Fig. 8A, C). In contrast, the end product from AD skin samples without PK-treatment exhibited an additional band migrating at approximately 48–50 kDa in addition to the four bands found in the end product of non-AD skin described above. This band could be oligomers of full-length or truncated tau molecules. In addition, the intensity of truncated trimer and dimer of tau migrating at 24–27 kDa was significantly increased compared to that of non-AD end product.

Upon PK-treatment, for the non-AD end products, the intensity of the two lower monomeric tau bands was significantly decreased while they were still detected at PK of 10 μg/mL (Fig. 8A, C). The intensity of the tau band migrating at ~ 25–27 kDa was increased first up to PK of 2.5 μg/mL and then decreased, until became undetectable at PK of 10 μg/mL. The band migrating at ~ 12 kDa seemed to be completely PK-sensitive and no band was detectable even at the lowest PK concentration at 1.25 μg/mL. In contrast, after PK-treatment the end product of the RT-QuIC with AD skin samples showed the decreased intensity of the tau band migrating at ~ 25–27 kDa but generated additional smaller band migrating at about 24 kDa (Fig. 8A, D, red arrow). This band was most likely derived from the truncation of the 25–27 kDa band since it was generated and increased over the increase in the PK concentration. In contrast with the non-AD end products, AD end product also exhibited an additional band between 7 kDa and 10 kDa bands migrating at about 8 kDa in the PK-treated AD skin end products (marked with the red arrow in Fig. 8A). The intensity of this band was similar to that of 7 kDa and 10 kDa bands and showed no changes upon the increase in the PK concentration (Fig. 8A, D).

Regarding the end product of sTau-SAA using 3RCF as the substrate, without PK-treatment, the AD and non-AD samples all mainly exhibited 3 bands migrating at about 7 kDa, 10–11 kDa, and 22–23 kDa on the gel (Fig. 8B). According to the sequence of the 3RCF molecule, the molecular weight of the monomeric 3RCF should be 10.5 kDa. Therefore, the 7 kDa band could be a truncated fragment of 3FCF while 22–23 kDa band could be a dimer of 3RCF. Since we got high intensity of the low molecular weight bands (~ 7 kDa), we increased the PK concentration to 25 μg/mL for 3R and decreased the loading amounts of samples (Fig. 8B, E). The intensity of the 3 bands from non-AD end products all decreased while there was a faint band emerging, migrating at approximately 5 kDa over the increase in PK concentrations. The intensity of the 3 bands from AD skin tau RT-QuIC end products was also all decreased while there were two additional bands emerging, migrating at approximately 16–18 kDa and 5–6 kDa over the increase in PK concentrations (Fig. 8B, F, red arrows). The monomers of truncated 4RCF (at ~ 12 kDa) and 3RCF (at ~ 10–11 kDa) exhibited no resistance to PK treatment. However, the dimers at higher molecular weights demonstrated increased resistance to PK, with the exception of the 4R negative end product dimers, which were digested at PK concentrations exceeding 7.5 μg/mL. Additionally, the low molecular weight bands below the monomers were more resistant to being digested within the chosen PK concentration range, especially with 4RCF.

### Conformational-stability assay of skin tau aggregates amplified by tau-SAA

We treated 4RCF (Fig. S9A) and 3RCF (Fig. S9B) tau-SAA end products with GdnHCl ranging from 0 to 3.2 M, followed by PK digestion at 10 μg/mL and quantitative analyses of GdnHCl/PK-resistant protein intensity of each treated sample. This approach is grounded on the principle that subtle differences in protein structure can be ascertained by assessing conformational stability when the protein is exposed to a denaturant such as GdnHCl at appropriate concentration ranges (23). In the absence of GdnHCl and PK, 3 tau bands migrating at 48 kDa, 25 kDa and 7–12 kDa were observed for 4RCF-based RT-QuIC end products, while 2 bands migrating at 22–23 kDa and 5–10 kDa were detected in 3RCF-based RT-QuIC end products. In contrast, both AD skin 4RCF-/3RCF-based tau RT-QuIC end products were found to have multiple or smear bands above 30 kDa (Fig. S9A, B). After GdnHCl and PK-treatment, there were virtually no PK-resistant tau bands detectable from both non-AD 4RCF/3RCF-based RT-QuIC end products. In contrast, positive skin tau RT-QuIC from AD participants with either 4RCF or 3RCF as the substrate showed PK-resistant tau fragments, especially for bands migrating at 25 kDa or lower for low concentration of GdnHCl (Fig. S9). Notably, there was an additional partially PK-resistant tau fragment migrating between 7 kDa and 5 kDa bands for 3RCF-based RT-QuIC end products, which was not detectable in the 4RCF-based skin tau RT-QuIC end products (Fig. S9). The GdnHCl concentrations required to make half of the tau end product sensitive to PK, referred to as GdnHCl1/2, were 2.6 M for positive 4R tau and 2.3 M for positive 3R tau for bands migrating at 22–25 kDa and 10 kDa, indicating that the positive 4R tau end product was approximately 1.13-fold more stable than the 3R tau end product. But, the 3RCF-based RT-QuIC end products from AD cases generated stable 7 kDa band (Fig. S9B, F).

## DISCUSSION

The demand for early and precise biomarkers in Alzheimer’s disease (AD) clinical practice has remained unmet. These biomarkers are essential not only for diagnosing and predicting the disease but also for facilitating patient participation in clinical trials and monitoring the effectiveness of therapeutic interventions [5]. With the recent positive outcomes from the Clarity AD trial [27] and the critical juncture in developing new strategies to prevent and slow down AD progression [28], the urgency for such biomarkers has intensified.

The recently developed AD-specific biomarkers utilized in clinical research have significantly enhanced our ability to diagnose and monitor AD pathology in living patients. These biomarkers have also provided valuable insights into the accumulation and spread of misfolded Aβ and tau aggregates in AD [29]. In both the initial 2018 and updated 2023 A/T/N research frameworks by the National Institute on Aging and the Alzheimer’s Association (NIA-AA), the detection of misfolded proteins, including Aβ and tau, through brain molecular imaging and body fluid analysis, has been central [30, 31]. A recent study assessing various brain imaging modalities in monitoring cognition and predicting cognitive decline has highlighted the significance of neocortical tau pathology as a key factor in cognitive decline over time, with tau-PET showing superior prognostic value compared to other neuroimaging measures [32]. An important advancement in the updated 2023 research framework is the inclusion of recently developed plasma biomarkers, supplementing the previously relied-upon biomarkers from cerebrospinal fluid (CSF) and brain imaging [30, 31]. However, despite these developments, there remain limitations associated with brain imaging, CSF, and Simoa-based plasma biomarkers, as outlined in the 2023 update [31]. It is also uncertain whether current biomarkers cover all relevant neuropathologies and fully reflect all aspects of pathogenesis. Moreover, the current list of AD biomarkers in the updated framework lacks markers capable of reflecting the pathological functions of misfolded proteins, such as the seeding activity of pathogenic tau or Aβ, as seen in the αSyn-SAA for PD. As a result, there is an ongoing quest for new minimally invasive or non-invasive biomarkers that can directly capture the unique pathogenic features of neurotoxic misfolded proteins.

Utilizing ultrasensitive RT-QuIC and/or PMCA techniques, our prior investigations have successfully identified minute quantities of misfolded proteins in skin samples obtained from individuals with PrD and PD by detecting their seeding activity, a prion-like characteristic of misfolded proteins. For example, we have illustrated that the seeding activity of PrPSc and pathogenic αSyn can be discerned in patients with Creutzfeldt-Jakob Disease (CJD), prion-infected rodents, and individuals affected by PD or other synucleinopathies, respectively [33–38, 8]. These findings have been corroborated by independent research groups [39–43]. Motivated by these discoveries, we have expanded our inquiry to encompass the most prevalent neurodegenerative condition, AD, and other tauopathies.

Our recent study has unveiled several groundbreaking discoveries. First, autopsy skin tissue from individuals with AD exhibited significantly higher levels of phosphorylated tau proteins detected by western blotting compared to non-AD tauopathies and normal controls. Moreover, the gel profile of the detected phosphorylated tau differed between AD and control groups, including other non-AD tauopathies and normal subjects. Second, similar to tau levels in AD brains [16, 19, 20], autopsy skin tau-SA detected by RT-QuIC was markedly higher in tauopathies than in normal controls, suggesting the potential use of sTau-SA as a novel diagnostic biomarker for tauopathies. Third, sTau-SA was detectable in cases with PD and DLB but not in MSA, although their seeding activity was significantly lower than that of AD and higher than normal controls. Fourth, autopsy skin tau aggregates from all tauopathies could seed the 3RCF substrate, whereas the 4RCF substrate could be seeded by skin tau aggregates from AD, PSP, and CBD but not from PiD. Fifth, the levels of skin tau-SA appeared to be associated with the progression of Braak staging observed in the brain. Sixth, biopsy skin tissues from individuals with AD and PSP showed significantly higher levels of tau-SA compared to normal controls, implying the potential of sTau-SA as a diagnostic biomarker for living tauopathy patients. Seventh, analysis of RT-QuIC end products revealed that sTau-SAA with AD skin samples formed tau oligomers and aggregates, confirmed by FTA, PK-treatment, conformational-stability assays, and TEM. ThT fluorescence intensity at the endpoint of the reaction correlated well with tau aggregate dot intensity by FTA. Eighth, PK-treatment of skin tau RT-QuIC end products in AD exhibited greater amounts of PK-resistant tau fragments than in normal controls. Finally, conformational-stability assays showed that skin tau could seed different strains in 4RCF and 3RCF substrates. These findings raise several important implications regarding the role of skin tau in the diagnosis and pathogenesis of tauopathies.

Studies have demonstrated an increase in tau gene expression in the skin of aging males [44]. Additionally, pathological tau deposits have been identified in peripheral organs such as the aorta, liver, spleen, and stomach of individuals with AD, but not in controls [45]. Notably, a distinct tau isoform known as big tau has been observed in peripheral tissues of both rodents and humans [46–49]. Phosphorylated tau has been detected in the skin of both AD participants and normal controls through various methods including immunohistochemistry, western blotting, and MALDI-MSI [26, 50–52]. Notably, most of the above studies revealed that the skin tau gel profile is different from that of brain tissues. Moreover, there have been inconsistencies in the gel profiles of skin tau observed in different studies [26, 51, 52]. For example, while some studies reported multiple tau bands in AD skin samples, others observed a single band or different tau band patterns [26, 48, 51–53]. Our own western blotting analysis revealed a distinctive skin tau gel profile with multiple bands in AD and other non-AD tauopathies, differing from previous observations [26, 51, 52]. These discrepancies could be attributed to variations in antibodies used, experimental conditions, and sample processing methods. This underscores the importance of standardizing experimental procedures and validating results across studies to better understand the role of skin tau in neurodegenerative diseases.

In addition to the previously reported bands at ~ 70 kDa, 55 kDa, and 45 kDa, our study revealed several additional bands migrating at approximately 110 kDa, ~ 25 kDa, and a high molecular weight band (HMWB) migrating above 110 kDa. The 110 kDa band may correspond to the big-tau isoform, as its molecular weight aligns with previous descriptions [46–49]. This band was consistently detected in all cases examined, including normal controls, except for one case with PiD pathology. Notably, the HMWB was present in cases from all groups except those with AD pathology. Furthermore, our analysis highlighted a distinct gel profile of tau bands in AD participants compared to other tauopathies and normal controls, with significantly elevated levels of all tau bands in AD. These findings suggest that skin tissue provides valuable insights into the levels and patterns of tau molecules, making it a promising specimen for investigating tau’s role in the pathogenesis of various tauopathies and for developing differential diagnostic tools to distinguish AD from other tauopathies and control subjects.

Our study is the first to demonstrate that tau extracted from both autopsy and biopsy skin samples of individuals with AD and other non-AD tauopathies exhibits significantly higher seeding activity compared to normal controls. This suggests that sTau-SA could serve as a novel diagnostic biomarker for tauopathies. Our 4RCF- or 3RCF-based RT-QuIC assay of autopsy skin samples from AD and non-AD tauopathies achieved a sensitivity of 75–80% and a specificity of 95–100%, respectively. Tau-SA was markedly elevated in biopsy skin samples from tauopathy patients compared to controls. Furthermore, our skin tau-based RT-QuIC assay holds promise for detecting the comorbidity of AD and PD. Both skin tau- or αSyn-SA can be detected depending on the substrate used [35–38, 54–56, current study]. Therefore, if a case exhibits both tau- and αSyn-pathology in the brain, our RT-QuIC assay is likely to detect both tau- and αSyn-SA in the skin, indicating comorbidity of AD and PD. However, reliable diagnosis of comorbidity requires ensuring that it does not result from cross-seeding between tau and αSyn, as tau-seeds can trigger αSyn aggregation and vice versa. Moreover, our findings regarding sTau-SA patterns shed light on the heterogeneity of tauopathies. Skin tau from PiD, characterized by 3R-dominated tau aggregates in the brain, seeded the 3RCF but not the 4RCF substrate in the RT-QuIC assay. In contrast, skin tau from AD, which features a mixture of 3R/4R tau, and from PSP/CBD, characterized by 4R-dominated tau aggregates in the brain, seeded both 4RCF and 3RCF substrates. These observations underscore the complexity of tauopathies and highlight the potential of skin tau seeding activity as a diagnostic tool to differentiate between different tauopathy subtypes.

Our investigation of skin-tau RT-QuIC end products confirmed that the increased tau-SA was accompanied by the formation of tau aggregates, as evidenced by our FTA, PK treatment, and conformational stability assays. TEM revealed the presence of oligomers and protofibrils, rather than mature fibrils, in the 3RCF- or 4RCF-based RT-QuIC end products of skin tau from AD cases, consistent with findings in the existing literature [57, 58]. Our previous study demonstrated that recombinant 4RCF fragments can spontaneously form mature fibrils in vitro within approximately two weeks [20]. Furthermore, analysis of PK-digested skin tau RT-QuIC end products showed the generation of additional PK-resistant tau fragments in positive RT-QuIC end products. Importantly, 3RCF- and 4RCF-based RT-QuIC end products generated different PK-resistant fragments, suggesting structural differences between the two. It will be intriguing to investigate whether the end products of tau-SAA with brain and skin samples yield similar or different structural and physicochemical features in future studies. This exploration could provide valuable insights into the pathogenic mechanisms underlying tau aggregation in both the central nervous system and peripheral tissues.

### Conclusions

In summary, our research highlights the promise of utilizing skin samples as a minimally invasive and readily accessible means for diagnosing tauopathies. Detecting phosphorylated tau and tau seeding activity in skin samples has the potential to advance novel diagnostic methods for these conditions. It is imperative to conduct additional studies involving larger patient groups and refine detection techniques to fully ascertain the clinical value of a skin-based diagnostic approach for distinguishing between various tauopathies.

## METHODS

### Design of the study

Skin samples were collected from two primary sources: 1) Cadavers with neuropathologically confirmed diagnoses of tauopathies and normal controls, and 2) Living patients with clinical diagnoses of AD and PSP. We performed RT-QuIC technique to examine the prion-like seeding activity of pathological tau. Statistical analyses were conducted to assess the sensitivity and specificity of the assay, utilizing different tau substrates to understand the impact on assay performance. All cadaveric skin samples were collected post-mortem and stored according to established protocols to preserve the biochemical properties of the tissue. Living patient samples were obtained through skin biopsies performed in a clinical setting and were immediately transported to the laboratory for analysis.

### Ethical statement

All procedures and protocols were monitored and approved by the Institutional Review Boards (IRBs) of University Hospitals Cleveland Medical Center, Banner Sun Health Research Institute, and IRCCS Institute of Neurological Sciences of Bologna. Written informed consent was obtained from all living subjects undergoing skin biopsy or from family members for skin autopsy. For post-mortem sample collection, we obtained the specimens with respect to the wishes of the deceased individuals and their families, following all legal and ethical guidelines. For skin biopsy procedures, all participants provided their informed consent prior to their inclusion in the study.

### Reagents and antibodies

Proteinase K (PK) and guanidine hydrochloride (GdnHCl) were purchased from Sigma Chemical Co. (St. Louis, MO, USA). Reagents for enhanced chemiluminescence (ECL Plus) were from Amersham Pharmacia Biotech, Inc. (Piscataway, NJ). Anti-tau mouse monoclonal antibodies RD3 and RD4 (Sigma-Aldrich) against human tau repeating region and sheep anti-mouse (SVM) IgG conjugated with horseradish peroxidase as a secondary antibody (AC111P, CHEMICON International, Inc, Burlington, MD) were used. Antibodies against Phospho-Tau (Thr231) and phospho-Tau (Ser396) were purchased from Cell Signaling Technology (Danvers, MA).

#### Source of skin samples

A total of 135 autopsy scalp skin samples from AD (n = 46), PSP (n = 33), CBD (n = 5), PiD (n = 6) and non-neurodegenerative controls (NNCs, n = 46) were collected and examined. These samples were obtained from the Arizona Study of Aging and Neurodegenerative Disorders (ASAND)/Brain and Body Donation Program at Banner Sun Health Research Institute through the Biomarkers across Neurodegenerative Diseases Research Grant 2019 (BAND 3) study. The diagnoses of these cases were confirmed via neuropathological examination of autopsied brain tissues at the ASAND. Biopsied skin samples from C7 paravertebral site (5 cm from the midline) of clinically diagnosed AD (n = 16), PSP (n = 8) and normal controls (n = 10) were from the Bellaria Hospital, Bologna, Italy, and the University Hospitals Cleveland Medical Center, Cleveland, Ohio, USA (see neuropathological and clinical information in Tables 1 and 2).

### Plasmid constructs cloning

Expression vectors for all six full-length wild-types human tau isoforms were generously provided by Dr. George Bloom of the University of Virginia (originated from the late Dr. Lester “Skip” Binder and Dr. Nicolas Kanaan of Michigan State University) (20, 59). 3RCF construct (three microtubule-binding repeats and cysteine-free construct containing C322S mutation) was first PCR-amplified of 3R repeats sequence from 2N3R tau plasmid and cloned into the same expression vector using Nde I and Xho I restriction sites, followed by site-directed mutagenesis at Cys322 site to Serine using QuikChange Site-directed mutagenesis kit (Agilent, Santa Clara, CA). 4RCF construct (four microtubule-binding repeats and cysteine-free construct containing C291S and C322S mutations) was first PCR-amplified of 4R repeats sequence from 2N4R tau plasmid and cloned into the same expression vector using Nde I and Xho I restriction sites, followed by site-directed mutagenesis at Cys291 and Cys322 sites using QuikChange Site-directed mutagenesis kit. All constructs were designed with a his6-tag at their carboxy-termini to facilitate protein purification and were verified by DNA sequencing.

### Engineered tau fragments 3RCF and 4RCF expression and purification

Recombinant 3RCF and 4RCF was prepared as previously described [20]. In brief, plasmids encoding human tau engineered constructs 3RCF and 4RCF were transformed into BL21-DE3 E. coli cells. Overnight starter cultures of BL21-DE3 E. coli cells transformed with recombinant tau plasmids were inoculated into multi-liter LB broth at 1:50 dilution and 100 mg/mL ampicillin. Cultures were incubated at 37°C, shaking until OD600 reached between 0.5 and 0.6. Tau expression was induced using 1 mM IPTG and continued to grow for an additional 4 hours. BL21-DE3 cells containing expressed tau were pelleted and resuspended in 50 mM NaH2PO4, pH 8.0 and 300 mM NaCl (sonication lysis buffer) at a concentration of 20 mL/L of culture preparation and sonicated at 60% power in ten 30-second intervals over 10 minutes. Cell lysates were centrifuged and supernatant containing the protein was applied to Ni-NTA column equilibrated with sonication lysis buffer. The columns were washed with 40–50 times of bed volumes of column buffer (sonication lysis buffer) followed by washing buffer (50 mM NaH2PO4, pH 8, 300 mM NaCl, and 20 mM imidazole). Recombinant protein was then eluted using elution buffer (50 mM NaH2PO4, pH 8, 300 mM NaCl, and 200 mM imidazole). Fractions were tested for protein concentration using 5 μL of protein sample mixed with 10 μL Coomassie Protein Assay reagent (ThermoFisher Scientific). Pooled fractions were concentrated to 4 mL using 10 kDa molecular weight cut-off spin columns (Millipore) and filtered using 0.22 μm low-binding Durapore PVDF membrane filters (Millipore). 3RCF and 4RCF tau proteins were further purified by FPLC using size exclusion Superdex-75 and Superdex-200 columns (GE Healthcare) in 1 x PNE buffer (25 mM PIPES, 150 mM NaCl and 1 mM EDTA, pH 7.0). Final 3RCF and 4RCF proteins were over 90% purity as evaluated by SDS-PAGE. Protein concentrations were quantified by BCA protein assays (ThermoFisher Scientific).

### Skin tissue preparation

Skin samples of approximately 30–100 mg in weight and 3–5 mm × 3–5 mm in size, primarily contained epidermis and dermis were collected as previously and prepared described [35, 37]. Briefly, skin tissues were homogenized at a 10% (w/v) concentration in a lysis buffer containing 2 mM CaCl2 and 0.25% (w/v) collagenase A (Roche) in Tris-Buffered Saline (TBS). The samples were incubated in a shaker at 37°C for 4 hours, shaking at 500 rpm, followed by homogenization using a Mini-BeadBeater (BioSpec, Laboratory Supply Network, Inc., Atkinson, NH).

### RT-QuIC Analysis

The RT-QuIC assay was modified as previously described with a slight modification [16, 18–20, 60]. In brief, the reaction mix for skin tau was prepared with 10 mM HEPES, pH 7.4, 200 mM NaCl, 10 μM ThT, and 10 μM either 4RCF or 3RCF tau substrate. In a 96-well plate (Nunc), 98 μL aliquots of the reaction mix were added to each well, followed by seeding with 2 μL of diluted skin homogenate (1:200 from 5% homogenate supernatant prepared by centrifugation at 3,000 g for 10 min at 4°C) in 10 mM HEPES, 1 x N2 supplement (Gibco), 1 x PBS and centrifuged at 5,000 g for 5 min at 4 °C. The plate was sealed with a plate sealer film (Nalgene Nunc International) and then incubated at 37°C in a BMG FLUOstar Omega plate reader. The incubation involved cycles of 1 min of orbital shaking followed by a 15-min of rest for the specified duration. ThT fluorescence measurements from bottom read (450 ± 10 nm excitation and 480 ± 10 nm emission) were recorded every 45 min. Each sample dilution contained 4 replicate reactions. The average ThT fluorescence values per sample were calculated using data from all four replicate wells, regardless of whether they crossed the threshold defined by ROC. A sample was considered positive if at least 2 of 4 replicate wells exceeded this threshold.

To quantify tau-SA detected by RT-QuIC, end-point dilution titrations were employed to determine the estimates of the sample dilution that generated positive reactions in 50% of the replicate reactions as the 50% seeding dose or SD50 (usually 2 out of 4 replicates) [15].

### Conformational stability immunoassay

The conformational stability immunoassay of RT-QuIC end products was conducted as previously described with a minor modification [61]. Briefly, 20 μL aliquots of end products were mixed with 20 μL of GdnHCl stock solution, resulting in final GdnHCl concentrations ranging from 0 to 3.0 M. After incubating at room temperature for 1.5 hours, samples were precipitated with a 5-fold volume excess of pre-chilled methanol overnight at −20°C. Following centrifugation at 14,000 g for 30 minutes at 4°C, the pellets were resuspended in 20 μL of lysis buffer (10 mM Tris-HCl, 150 mM NaCl, 0.5% Nonidet P-40, 0.5% deoxycholate, 5 mM EDTA, pH 7.4). Each aliquot was digested with 10 μg/mL PK for 30 minutes at 37°C. The reaction was terminated with cOmplete protease inhibitor cocktail (CO-RO, Roche), and the samples were boiled in SDS loading buffer and loaded onto 15% Tris-HCl pre-cast gels (Bio-Rad) for Western blotting analysis.

### Western blotting

The samples prepared as described above were separated using 15% Tris-HCl Criterion pre-cast gels (Bio-Rad) in SDS-PAGE. Proteins from the gels were transferred onto Immobilon-P polyvinylidene fluoride (PVDF, Millipore) membranes for 90 minutes at 70 V. To probe with the anti-tau antibodies (RD3, RD4, pT231, or pS396), the membranes were incubated overnight at 4°C with a 1:1,000–1:4,000 dilution of the primary antibodies. After incubation with a 1:4,000–1:5,000 dilution of horseradish peroxidase-conjugated sheep anti-mouse IgG, tau bands were visualized on Kodak film using ECL Plus as instructed by the manufacturer. Densitometric analysis was used to measure the intensity of tau protein bands, which were quantified with UN-SCAN-IT Graph Digitizer software (Silk Scientific, Inc., Orem, Utah).

### Filter-trap assay

The filter-trap assay was used to determine the RT-QuIC reaction mixtures with increased ThT fluorescence formed aggregates and to evaluate their sizes as described previously [62]. In brief, the end products of RT-QuIC were mixed with washing buffer containing 2% SDS, 10 mM Tris-HCl, pH 8.0 and 150 mM NaCl for an hour at room temperature. Following incubation, the samples were filtered through a cellulose acetate membrane (Advantec MFS, Dublin, CA). After filtering, the membrane was rinsed with washing buffer to remove unbound proteins and subsequently blocked with 5% BSA in 0.1% Tween-20 in 1 x PBS for an hour. The membrane was then probed with RD3 and RD4 antibodies, followed by incubation with sheep anti-mouse secondary antibody. The proteins on the membrane were visualized using ECL Plus, and the resulting signal was captured using a chemiluminescent imaging system with X-ray/automatic film processor. Densitometric analysis was used to measure the intensity of tau protein dots for the quantitative analysis as mentioned above.

### Transmission electron microscopy

Transmission electron microscopy (TEM) images were collected as previously described [20, 63]. Briefly, the skin tau-SAA end product samples at a concentration of 10 μM were maintained in a frozen state until ready for TEM analysis. Before imaging, 2 μL of the sample was applied to a 200 mesh formvar-carbon coated grid and allowed to sit for 5 minutes. The excess sample was gently removed using filter paper. A thorough examination of each grid was conducted to qualitatively assess the presence of oligomers or fibrils. Representative images were taken from 15–20 distinct locations on each grid. The TEM studies were conducted using a JEOL-1400 transmission electron microscope (JOEL United States, Inc., Peabody, MA) at an operating voltage of 120 kV.

### Statistical analysis

Experimental data were analyzed using Student’s t-test for comparing two groups. McNemar’s test was employed to assess marginal homogeneity and differences in agreement. For comparisons between PD versus CBD and PSP, where the sample size allowed, we conducted a paired area under the ROC curve (AUC) analysis to evaluate significant differences in AUC values. Tests adopted a two-sided type II error level of 0.05.

## Figures and Tables

**Table 1:** Demographic and neuropathological features of autopsied cases in different groups

Neuropathological diagnosis	No. cases	Sex (M/F)	Age (years, mean ± SD)	Post-mortem Interval (hours, mean ± SD)	Braak score	Plaque total (mean ± SD)	Tangle total (mean ± SD)	Comorbidities
Alzheimer’s disease	46	18/28	76.25 ± 8.67	7.08 ± 2.93	43% IV, 13% V, 44% VI	14.1 ± 1.18	13.86 ± 2.21	4/46 with TDP-43 proteinopathy
Corticobasal degeneration	5	3/2	72.5 ± 8.20	5.74 ± 2.13	100% V	3.80 ± 5.84	12.90 ± 0.42	1/5 with AD
Progressive supranuclear palsy	33	23/10	82.73 ± 11.90	6 ± 2.77	15% III, 60% IV, 25% V	4.74 ± 5.69	7.67 ± 2.39	4/33 with Lewy bodies by Unified LB Stage
Pick’s disease	6	2/4	71.5 ± 7.66	7.5 ± 1.48	33% II, 67% III	3.33 ± 4.14	4.08 ± 3.43	1/6 with AD
Parkinson’s Disease	10	3/7	80.1 ± 6.87	4.67 ± 1.82	60% II, 30% III, 10% IV	3.98 ± 4.98	3.32 ± 1.95	None
Dementia with Lewy Bodies	6	3/3	77.17 ± 5.34	2.80 ± 1.64	17% I, 17% II, 33%IV, 33%VI	12.54 ± 2.15	8 ± 5.47	4/6 with AD
Multiple system atrophy	6	4/2	71.67 ± 7.53	3.27 ± 0.85	33% I, 17%II, 33% III, 17% IV	4.96 ± 5.71	3.08 ± 2.22	None
Non-neurodegenerative Controls	43	27/16	79.44 ± 14.19	0	37% I, 28% II, 35% III	1.44 ± 2.13	2.69 ± 1.62	None

**Table 2:** Demographic and clinical features of biopsied cases in different groups

Clinical diagnosis	Number of cases	Sex (M/F)	Age (yrs., mean ± SD)	Disease duration (yrs., mean ± SD)	MMSE (mean ± SD)	Hoehn & Yahr stage (mean ± SD)
Alzheimer’s disease	16	6/10	70.13 ± 9.68	3.04 ± 2.14	19.35 ± 5.44	0
Progressive supranuclear palsy	8	5/3	75.71 ± 9.36	6 ± 2.77	25.67 ± 4.93	3.27 ± 1.44
Non-neurodegenerative disease	10	3/7	68.80 ± 10.83	0	Not available	0

**Table 3:** Demographic and clinical features of individual biopsied cases in different groups

Clinical diagnosis	Age	Sex	Disease duration (years)	Hoehn & Yahr stage	MMSE	MoCA	Cohort	Biopsy position
AD1	71	F	3	0	19	N/A	Italy	lateral to C7
AD2	81	M	2	0	22	N/A	Italy	lateral to C7
AD3	80	M	1	0	10	N/A	Italy	lateral to C7
AD4	78	F	4	0	14	N/A	Italy	lateral to C7
AD5	65	M	3	0	12	N/A	Italy	lateral to C7
AD6	55	M	5	0	24	N/A	Italy	lateral to C7
AD7	54	M	2	0	26	N/A	Italy	lateral to C7
AD8	61	M	2	0	24	N/A	Italy	lateral to C7
AD9	81	F	1	0	25	N/A	Italy	lateral to C7
AD10	83	F	3	0	16	N/A	Italy	lateral to C7
AD11	63	F	2	0	24	N/A	Italy	lateral to C7
AD12	76	F	8	0	N/A	N/A	Italy	lateral to C7
AD13	65	F	3	0	28	N/A	Italy	lateral to C7
AD14	62	F	2	0	24	N/A	Italy	lateral to C7
AD15	71	F	7	0	17	N/A	Italy	lateral to C7
AD16	71	F	1	0	14	N/A	Italy	lateral to C7
PSP1	72	M	1	2	20	N/A	Italy	lateral to C7
PSP2	65	F	3	2	28	N/A	Italy	lateral to C7
PSP3	59	F	2	1	29	N/A	Italy	lateral to C7
PSP4	88	F	2	4	20	21	Cleveland	lateral to C7
PSP5	84	M	7	5	20	25	Cleveland	lateral to C7
PSP6	75	M	4	2.5	70	15	Cleveland	lateral to C7
PSP7	76	M	5	4	30	21	Cleveland	lateral to C7
PSP8	79	M	4	4	40	28	Cleveland	lateral to C7
NC1	59	F	N/A	0	N/A	26	Cleveland	lateral to C7
NC2	69	M	N/A	0	N/A	27	Cleveland	lateral to C7
NC3	72	F	N/A	0	N/A	28	Cleveland	lateral to C7
NC4	63	F	N/A	0	N/A	26	Cleveland	lateral to C7
NC5	81	F	N/A	0	N/A	25	Cleveland	lateral to C7
NC6	71	M	N/A	0	N/A	28	Cleveland	lateral to C7
NC7	73	F	N/A	0	N/A	27	Cleveland	lateral to C7
NC8	74	F	N/A	0	N/A	25	Cleveland	lateral to C7
NC9	77	F	N/A	0	N/A	30	Cleveland	lateral to C7
NC10	77	M	N/A	0	N/A	23	Cleveland	lateral to C7

* N/A: Not applicable.

## Data Availability

All data are available in the main text or the supplementary materials.

## Abbreviations

αSyn: α-synuclein
AD: Alzheimer’s disease
CJD: Creutzfeldt-Jakob disease
CSF: Cerebrospinal fluid
CBD: Corticobasal degeneration
DLB: Dementia with Lewy bodies
FTA: Filter-trap assay
GdnHCl: Guanidine hydrochloride
HMWB: High molecular weight band
PrPSc: Infectious prion protein
MSA: Multiple system atrophy
NIA-AA: National Institute on Aging and the Alzheimer’s Association
PD: Parkinson’s Disease
PiD: Pick’s disease
PrD: Prion disease
PK: Proteinase K
PMCA: Protein misfolding cyclic amplification assay
PSP: Progressive supranuclear palsy
RT-QuIC: Real-time quaking-induced conversion
SAA: Seed-amplification assay
SA: Seeding activity
SVM: Sheep anti-mouse
Simoa: Single molecular immunoassay
TEM: Transmission electron microscopy
